# Hexokinase II inhibitor, 3-BrPA induced autophagy by stimulating ROS formation in human breast cancer cells

**DOI:** 10.18632/genesandcancer.9

**Published:** 2014-03

**Authors:** Qianwen Zhang, Yuanyuan Zhang, Pei Zhang, Zhenhua Chao, Fei Xia, Chenchen Jiang, Xudong Zhang, Zhiwen Jiang, Hao Liu

**Affiliations:** ^1^ Faculty of pharmacy, Bengbu Medical College, Bengbu, Anhui, P. R. China; ^2^ School of Medicine and Public Health, Faculty of Health, University of Newcastle, NSW, Australia

**Keywords:** 3-BrPA, CQ, Autophagy, Apoptosis, Necroptosis, ROS

## Abstract

Hexokinase II (HKII), a key enzyme of glycolysis, is widely over-expressed in cancer cells. 3-bromopyruvate (3-BrPA), an inhibitor of HK II, has been proposed as a specific antitumor agent. Autophagy is a process that regulates the balance between protein synthesis and protein degradation. Autophagy in mammalian systems occurs under basal conditions and can be stimulated by stresses, including starvation, oxidative stress. Therefore, we hypothesized that 3-BrPA could induce autophagy. In the present study, we explored the mechanism of 3-BrPA and its combined action with chloroquine. Our results demonstrate that in MDA-MB-435 and in MDA-MB-231 cells, 3-BrPA induces autophagy, which can be inhibited by chloroquine. Furthermore, the combined treatment synergistically decreased the number of viable cells. Interestingly, the combined treatment triggered apoptosis in MDA-MB-435 cells, while it induced necroptosis in MDA-MB-231 cells. ROS mediated cell death when 3-BrPA and CQ were co-administered. Finally, CQ enhanced the anticancer efficacy of 3-BrPA *in vivo*. Collectively, our results show that 3-BrPA triggers autophagy, increasing breast cancer cell resistance to 3-BrPA treatment and that CQ enhanced 3-BrPA-induced cell death in breast cancer cells by stimulating ROS formation. Thus, inhibition of autophagy may be an innovative strategy for adjuvant chemotherapy of breast cancer.human skeletal muscle. Efficient Mirk depletion in SU86.86 pancreatic cancer cells by an inducible shRNA decreased expression of eight antioxidant genes. Thus both cancer cells and differentiated myotubes utilize Mirk kinase to relieve oxidative stress.

## INTRODUCTION

Cancer cells undergo increased aerobic glycolysis, increasingly relying on this metabolic pathway to generate ATP to sustain elevated proliferation. That an impaired glucose metabolism leads to lactic acid secretion in the presence of oxygen was first described by Warburg in the 1920s [1]. This increased aerobic glycolysis has since been observed in many tumor cells. Consequently, specific drugs designed to interfere with energy-producing pathways of many cancer types have been investigated [2,3], such as the glycolytic inhibitors 2-deoxyglucose [4], and 3-BrPA [5]. 3-BrPA is an analogue of pyruvate with high tumor selectivity [6,7]. In mammals, there are four hexokinase isoforms, HK1, HK2, HK3, and HK4, encoded by separate genes. HK2 is expressed at relatively high level only in skeletal muscles, adipose tissues, and heart. Despite its absence or low expression in the majority of adult normal cells, HK2 is widely over-expressed in many cancer cells [8]. Patra's group showed that HK2 expression is dramatically elevated in tumors derived from mouse models of lung and breast cancer [9]. Cecilia's group found that HK I and II inhibition by metformin could modify glucose metabolism in triple-negative breast cancer both in cultured cells and xenograft models [10]. So it seemed feasible that HK2 could be a selective therapeutic target for cancer. 3-BrPA can not only inhibit HKII and prevent glucose from entering the glycolytic pathway [11], but the compound can also induce cell death by activating the mitochondrial pathway of apoptosis or necrosis [12,13].

Programmed cell death is executed through specific intracellular biochemical pathways. Apoptosis, autophagy, necrosis and/or necroptosis are three common forms of programmed cell death distinguished according to their morphological, enzymological and functional criteria. Apoptosis is executed by a group of intracellular cysteine proteases, namely caspases. Necrosis, on the other hand, is associated with organelle swelling, cytoplasmic membrane breakdown, and ensuing inflammation responses [14]. Necroptosis is a regulated necrotic cell death triggered by broad caspase inhibition and is characterized by necrotic cell death morphology and the activation of autophagy [15]. Receptor-interacting protein kinase 1 (RIP1) kinase activity is then crucial for this pathway, which may also be mediated via the Fas, TNF or TNF-related apoptosis-inducing ligand (TRAIL) death receptors [16]. Autophagy is a major intracellular pathway for the degradation and recycling of unused but long-lived proteins, damaged organelles, and even invasive pathogens [17,18]. Some studies have highlighted the importance of autophagy in several organs, including the brain, heart, hematopoietic cells, and the kidney [19,20,21,22]. For instance, systemic autophagy-knockout mice die within one day after birth [23]. Although autophagy is important for normal cell function and survival, it is also used by tumor cells and may be therapeutically counterproductive [24,25]. Activation of autophagy has been suggested to promote cell survival [26], so a combination chemotherapeutic treatment with an autophagy inhibitor could promote tumor cell death [27]. Chloroquine (CQ) is an anti-malarial drug, as well as a well-known lysosomotropic agent that inhibits late-stage autophagy [28]. An analogue of CQ, hydroxychloroquine (HCQ), has also been shown to have antitumor properties [29]. HCQ promotes tumor cell death though either p53 activation or alkylation in a mouse model of c-Myc-driven lymphoma [30]. However, the precise mechanism by which autophagy inhibition promotes cancer cell death remains to be determined, particularly with respect to glycolytic inhibitors and the definition of cancer susceptibility to autophagy inhibition.

The role of autophagy in 3-BrPA-induced cell death in human breast cancer cells has not, to our knowledge, been explored. Thus, we investigated whether autophagic machinery could be activated in breast cancer cells after 3-BrPA treatment and whether inhibition of autophagy enhanced 3-BrPA-mediated cell death. Furthermore, we demonstrated that ROS formation increased cell sensitivity to death via autophagy inhibition. Finally, we demonstrated that inhibition of autophagy enhanced 3-BrPA-mediated cell death *in vivo*.

## RESULTS

### The effects of 3-BrPA on cell proliferation in breast cancer cell lines MDA-MB-435 and MDA-MB-231

**Figure 1:**
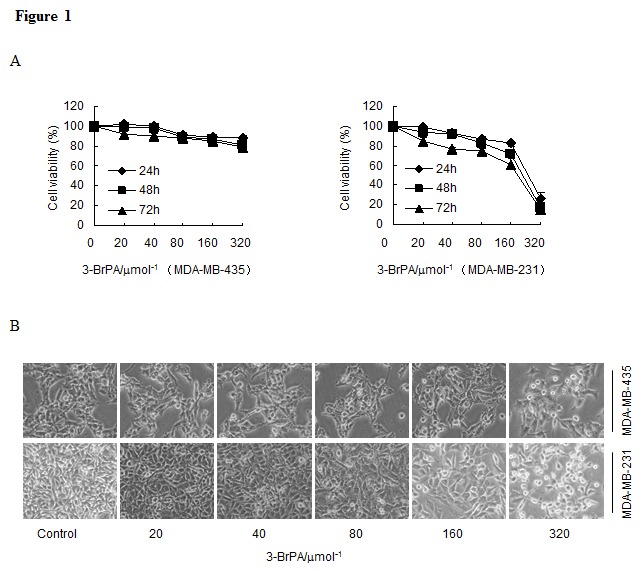
3-BrPA affects the cell viability of breast cancer cells (A) Cells seeded on 96-well plates (5×10^3^ cells/well) were treated with the indicated concentrations of 3-BrPA for 24, 48, and 72 h. Then, relative cell viability was assessed using an MTT assay. (B) After incubation with the indicated concentrations of 3-BrPA, cells were photographed using an inverted microscope.

As an analogue of pyruvate, 3-BrPA can induce cell death in certain tumor cell lines. To investigate 3-BrPA's potential to inhibit cell growth in breast cancer cells (MDA-MB-231, MDA-MB-435), cell viability was measured with an MTT assay after incubation of each cell line with 3-BrPA for various periods. As shown in Figure 1A, 3-BrPA (0–320 μM) reduced MDA-MB-231 cell growth in a dose- and time- dependent manner. 3-BrPA had little effect on MDA-MB-435 cell growth (Figure 1A). As visualized with an inverted microscope, viable MDA-MB-231 cells decreased significantly and dose-dependent manner after 24 h of 3-BrPA treatment. Most cells shrank and became rounded before detaching from the culture plates (Figure 1B), whereas viable MDA-MB-435 cells only modestly decreased. These data prompted us to explore the unique effects of 3-BrPA on MDA-MB-435 and MDA-MB-231 cells.

### 3-BrPA induces autophagy in breast cancer cells

**Figure 2:**
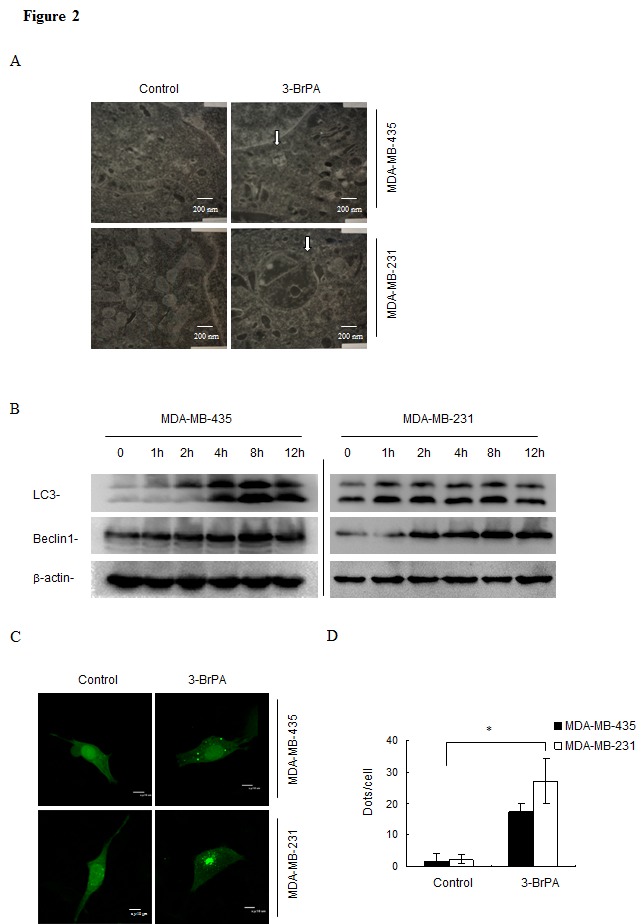
3-BrPA induces autophagy in breast cancer cells (A) Breast cancer cells MDA-MB-435 and MDA-MB-231 were treated with 320μM 3-BrPA and 160 μM 3-BrPA respectively for 12 h as indicated. Then cells were collected and prepared for electron microscopy (EM) analysis as described in materials and methods. The arrows indicate the appearance of autophagosomes (scale bar = 200 nm). (B) Cells were treated as above for 0, 1, 2, 4, 8, and 12 h as indicated, and the extracted protein was immunoblotted against LC3 and Beclin-1 antibody. β-actin was used to normalize the data for equal protein loading. (C) Cells were transiently transfected with GFP-tagged LC3 plasmid DNA (GFP-LC3), treated with 3-BrPA (320 μM) and 3-BrPA (160 μM) as above for 12 h, then subjected to confocal microscopy analysis (scale bar = 10μm). Quantification of LC3 punctation of three independent experiments are shown in panel (D) *, *p*< 0.05 versus control at a given time point.

First electron microscopy (EM) was used to visualize cell morphology after both cell lines were treated with 3-BrPA. As shown in Figure 2A, 3-BrPA treatment increased the presence of autophagosomes filled with debris in both cell lines; only a few vacuoles were observed in control cells. Autophagy-specific markers such as microtubule-associated protein 1 LC3, Beclin-1 were used to quantify autophagy with immunoblot analysis. As shown in Figure 2B, conversion of LC3 I/II and up-regulation of Beclin-1 suggested increased formation of autophagosomes in a time-dependent manner in breast cancer cells. Induction of autophagy by 3-BrPA was then examined by imaging the cellular distribution of GFP-LC3, a fusion construct of green fluorescent protein with LC3. In control cells, GFP-LC3 puncta were mainly distributed to the cytosol, indicating a low level of autophagy under these conditions (Figure 2C, left panel). In contrast, cells incubated with 3-BrPA for 12 h revealed many autophagosomes with accumulated GFP-LC3 (presumably as GFP-LC3-II). Quantification data for puncta per cell are depicted in Figure 2D. These data suggest that 3-BrPA induces a complete autophagic response in breast cancer cells.

### Autophagy inhibition enhances 3-BrPA-induced cell death

**Figure 3:**
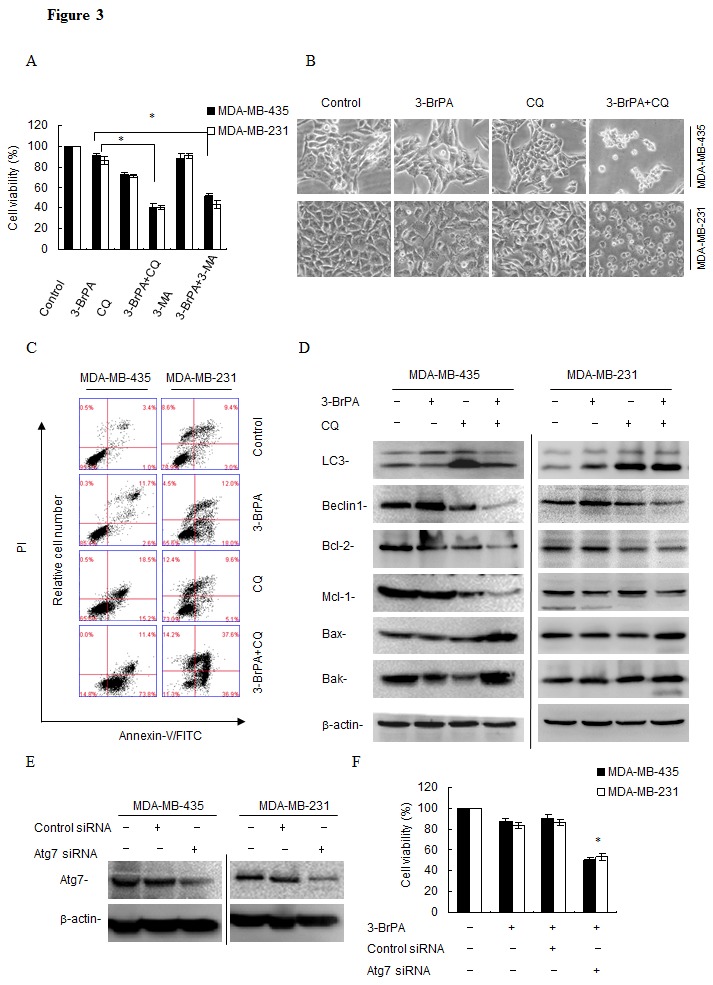
Autophagy inhibition enhances 3-BrPA-induced cell death *in vitro* (A) After pre-incubation with 40 μM CQ/5 mM 3-MA for 1 h, MDA-MB-435 cells and MDA-MB-231 cells were treated with 320 μM 3-BrPA and 160 μM 3-BrPA respectively for 12 h, and the relative cell viability was assessed with an MTT assay. (B) Cells were treated as above. Then cells were photographed using an inverted microscope. (C) After pre-incubation with 40 μM CQ for 1 h, cells were treated as above, then stained with Annexin V-FITC/PI and measured using flow cytometry. (D)After cells were treated as above, and cell lysates were prepared, then subjected to western blot analysis to measure LC3 and Beclin-1. Lysates were also assayed to measure Bak, Bax, Bcl-2 and Mcl-1. β-actin was used to normalize the data for equal protein loading. Data represent 2 independent experiments. (E) Cell lysates were collected 48 h posttransfection and knockdown efficiency of Atg7 RNAi was assessed using western blot quantification of Atg7 and β-actin. (F) Cells were transfected with the control or Atg7 siRNAs. Then, after 48 h posttransfection, MDA-MB-435 cells and MDA-MB-231 cells were treated with 320 μM and 160 μM 3-BrPA respectively, and/or with 40 μM CQ for 12 h, and relative cell viability was assessed with an MTT assay. Data are represented as mean ±standard deviation of duplicates. *, *p*<0.05 versus control at a given time point.

To investigate the role of autophagy in cell death, we inhibited autophagy with CQ or 3-Methyladenine (3-MA) and measured cell death. As expected, a significant increase in 3-BrPA-induced cell death was observed in breast cancer cells after autophagy was inhibited with CQ or 3-MA (Figure 3A). As viewed with an inverted microscope, viable MDA-MB-231 and MDA-MB-435 cells were significantly decreased after 3-BrPA plus CQ treatment for 12 h. The majority of cells shrank and became round before detaching from the culture plates (Figure 3B). Moreover, annexin V-propidium iodide staining revealed a significant increase in cell death (defined as annexin V+ and PI V+cells) after 3-BrPA plus CQ treatment (Figure 3C).

To ascertain whether 3-BrPA plus CQ affects autophagy, we measured expression of autophagy-related proteins using western blot. Accumulation of LC3-II indicates high flux through the autophagy pathway, 3-BrPA increased turnover and clearance of LC3-positive autophagosomes and LC3-II as indicated by western blot and increased autophagic flux blocked was with CQ. Accordingly, the concentration of Beclin1 was reduced by 3-BrPA in the presence of CQ (Figure 3D). Examining apoptosis-related proteins, we discovered that 3-BrPA plus CQ treatment significantly inhibited anti-apoptotic Bcl-2 and MCl-1 expression and enhanced pro-apoptotic Bax and Bak expression (Figure 3D). Knockdown of Atg7 siRNA also sensitized breast cancer cells to 3-BrPA, indicating that autophagy was responsible for 3-BrPA resistance (Figure 3E and 3F). These data suggest that autophagy is a mechanism of resistance to 3-BrPA that could be reversed by genetic blockade of autophagy initiation or pharmacologic blockade of cargo degradation.

### 3-BrPA plus CQ treatment induces RIPK1-dependent apoptosis in MDA-MB-435 cells and RIPK1/3-dependent necroptosis in MDA-MB-231 cells

**Figure 4:**
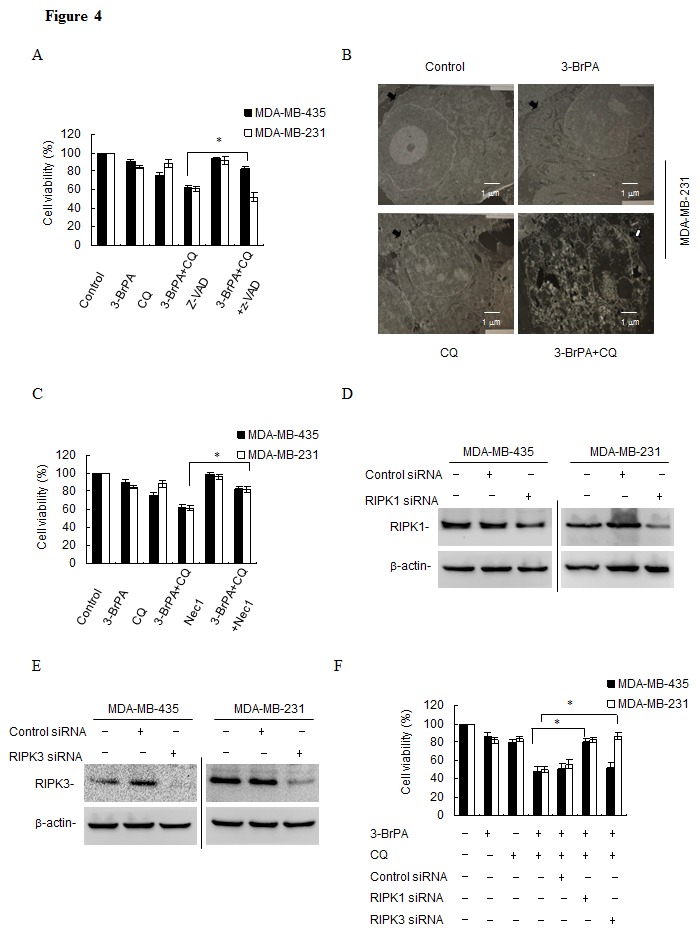
3-BrPA and CQ co-operate to induce RIPK1-dependent apoptosis in MDA-MB-435 cells, but induce RIPK1/3-dependent necroptosis in MDA-MB-231 cells (A) After pre-incubation with 10 μM z-VAD-fmk for 1 h, cells were treated with 160 μM 3-BrPA and/or with 20 μM CQ for 24 h. Then, relative cell viability was assessed with an MTT assay. (B) MDA-MB-231 cells were treated with 160 μM 3-BrPA and/or with 20 μM CQ for 24 h, and then cells were collected and prepared for EM analysis. Black arrowheads denote cell membrane integrity in DMEM-treated cells and membrane breakdown in cells treated with 3-BrPA plus CQ. White arrowheads denote the swelling of cellular organelles in cells treated with 3-BrPA plus CQ (scale bar = 1 μm). (C) After pre-incubation with 10 μM Nec-1 for 1 h, MDA-MB-435 and MDA-MB-231cells were treated with 160 μM 3-BrPA and/or with 20 μM CQ for 24 h. Then, relative cell viability was assessed with an MTT assay. (D) Cell lysates were collected 48 h posttransfection, and knockdown efficiency of RIPK1 (RIP1) RNAi was assessed by measuring RIP1 and β-actin with western blot. (E) Cell lysates were collected 48 h posttransfection and knockdown efficiency of RIPK3 (RIP3) RNAi was assessed using Western blot analysis to measure RIP3 and β-actin. (F) Cells were transfected with control or RIP1/RIP3 siRNAs. Then, 48 h posttransfection, cells were treated with 160 μM 3-BrPA and/or with 20 μM CQ for 24 h. Relative cell viability was assessed with an MTT assay. Data are represented as mean±standard deviation of duplicates. *, *p*< 0.05 versus control at a given time point.

To confirm the cell death type induced by the combination of 3-BrPA and CQ, we used a broad spectrum caspase inhibitor, z-VAD-fmk to rescue cells from death induced by 3-BrPA plus CQ treatment. As shown in Figures 4A, an MTT assay revealed that z-VAD-fmk rescued MDA-MB-435 cells, but exacerbated cell death in MDA-MB-231 cells. Using EM, we observed that 3-BrPA plus CQ-treated MDA-MB-231 cells had ruptured plasma membranes, indicating necrosis (Figure 4B). Death may also occur by a programmed form of necrosis, necroptosis, which requires RIPK1 and RIPK3 activation by death receptors [31] or other death receptor-independent mechanisms [32]. To assess whether 3-BrPA plus CQ treatment induced necroptosis, MDA-MB-231 cells were treated with 3-BrPA plus CQ with/without the specific RIPK1 and necroptosis inhibitor necrostatin-1 (Nec-1). Nec-1 alone had no effect on cells, but dramatically restored cell survival by after treatment with 3-BrPA plus CQ in MDA-MB-231 cells, and it also had the same effect on MDA-MB-435 cells (Figures 4C). Knockdown of RIPK1 in both cell lines (Figure 4D) also suppressed cell death and enhanced cell viability (Figure 4F). Similarly, MDA-MB-231 cells treated with a validated siRNA against RIPK3 (Figure 4E) were significantly more viable after treatment with 3-BrPA plus CQ, while knockdown of RIPK3 had no effect on MDA-MB-435 cells (Figure 4F). These data suggest that 3-BrPA and CQ induce two different cell death types in MDA-MB-435 cells and MDA-MB-231 cells, and both death pathways require RIPK1.

### ROS production causes cell death with 3-BrPA and autophagy inhibition

**Figure 5:**
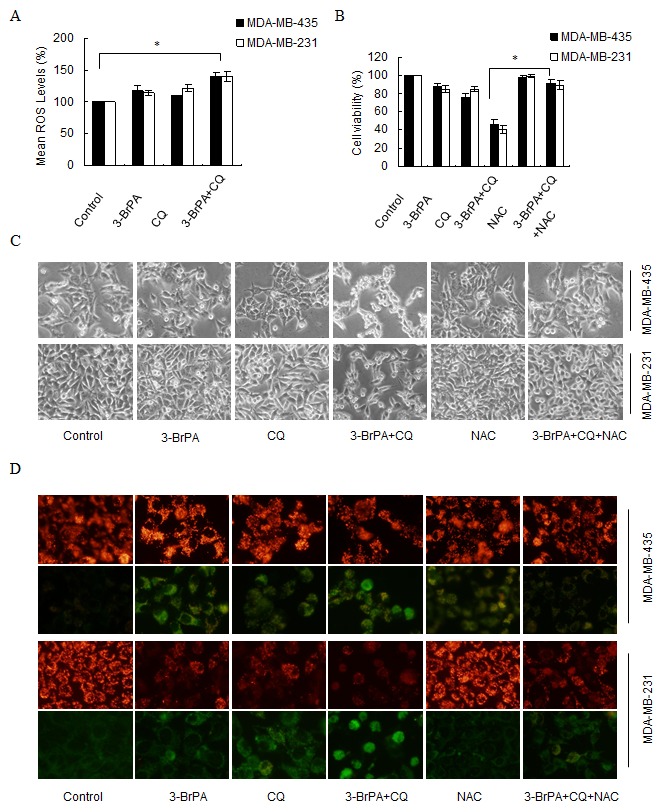
ROS production contributes to 3-BrPA- and CQ-induced cell death in breast cancer cells (A) MDA-MB-435 cells and MDA-MB-231 cells were treated with 320 μM 3-BrPA and 160 μM 3-BrPA respectively, and/or with 40 μM CQ for 12 h. ROS production was analyzed with DHE staining and flow cytometry. Mean ROS (100%) values are shown. Experiments were performed in triplicate, *, *p*< 0.05 versus control at a given time point. (B) After pre-incubation with 5 mM NAC for 1 h, cells were treated as above. Relative cell viability was measured with an MTT assay. (C) After pre-incubation with 5 mM NAC for 1 h, cells were treated as above. Cells were photographed using an inverted microscope. (D) Cells were treated as above. Loss of mitochondrial membrane potential (MMP) was quantified with JC-1 staining and fluorescent microscopy.

Next, to elucidate the mechanism which leads to the initiation of 3-BrPA-related autophagy, we quantified intracellular ROS in breast cancer cells after a 12 h incubation with 3-BrPA, CQ, or a combination of both drugs. As shown in Figure 5A, ROS generation was markedly increased in cells treated with 3-BrPA plus CQ compared to cells treated with 3-BrPA alone. Because elevated ROS occurred in cells with suppressed autophagy, we investigated whether ROS inhibition influenced 3-BrPA-plus-CQ-mediated cell death. ROS formation was inhibited with the ROS scavenger N-acetyl cysteine (NAC) and cell viability was measured with an MTT assay. Cell morphology was observed and photographed under an inverted microscope (Figures 5B, 5C). Our data indicate that cell sensitization to 3-BrPA- and CQ-induced completely blocked cell death when ROS formation was inhibited. In addition, the mitochondrial membrane potential (MMP), an indicator of ROS generation, was detected in these cells using JC-1 [33]. Higher MMP was observed in the control culture group compared to the dually treated groups (Figure 5D). With co-treatment with 3-BrPA and CQ, JC-1 revealed higher green fluorescence, while adding NAC makes MMP turn to be higher again. (Figure 5D).

### CQ enhanced anti-tumor efficacy of 3-BrPA in nude mice

**Figure 6:**
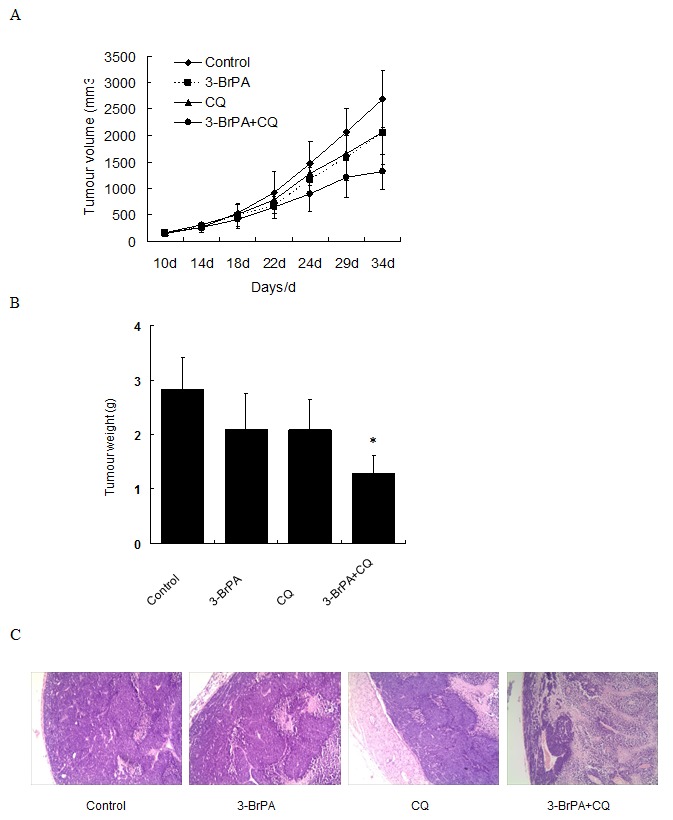
CQ enhanced the anti-tumor ability of 3-BrPA in nude mice Human MDA-MB-231 cells were inoculated subcutaneously to form tumors. Mice with tumors (100–200 mm^3^) were randomized into four groups (5 mice/group). Vehicle (0.9% NS) or CQ (40 mg/kg/d) or 3-BrPA (5 mg/kg/d) alone or in combination was administrated to the mice intraperitoneally once a day. (A) Tumor growth was monitored every three days and calculated using the formula: length*width^2^/2. (B) The tumor weights of mice after 3-BrPA and CQ-treatment. (C) HE staining *in vivo*.

To ascertain whether a combination of 3-BrPA and CQ could suppress tumor growth, MDA-MB-231 xenografts were examined. Tumors continued to grow in xenografted mice treated with vehicle, CQ, or 3-BrPA, or a combination of CQ and 3-BrPA. The combination of CQ plus 3-BrPA prevented tumor growth (Figure 6A). After treatment ended, mice were sacrificed, and tumors were excised and evaluated. Tumor weights of mice treated with vehicle, CQ, and 3-BrPA alone were greater than those from mice treated with CQ plus 3-BrPA (Figure 6B). Hematoxylin and eosin (HE) staining indicated greater necrotic areas in CQ plus 3-BrPA-treated mice. Thus, CQ enhanced the anti-tumor efficacy of 3-BrPA *in vivo*.

## DISCUSSION

The present study demonstrates that exposure of breast cancer cells to 3-BrPA induces autophagy and that CQ enhances the anti-tumor efficacy of 3-BrPA, both *in vitro* and *in vivo*. The lethal effect of 3-BrPA in combination with CQ is initially triggered by the development of ROS-dependent autophagy induced by 3-BrPA, and then inhibition of autophagy and subsequent induction of mitochondrial depolarization and more ROS production, resulting in two different forms of cell death in MDA-MB-435 cells and MDA-MB-231 cells.

The roles of autophagy within the context of cancer are controversial. One view is that autophagy suppresses tumor progress; another view is that autophagy is a pro-survival pathway. Inactivation of the autophagy-specific gene, Beclin-1, produces significantly higher frequencies of spontaneous malignancies in mice [34]. Muhammad's group showed that palmitic acid triggers Ca2+-dependent autophagy, which results in programmed necrotic death (necroptosis) in endothelial cells [35]. However, autophagy may also be a pro-survival pathway assisting tumor cells to endure metabolic stress under nutrient/oxygen-deficient conditions and resist death triggered by chemotherapeutic agents. Xu's group showed that accumulation of autophagosomes in breast cancer cells induced

TRAIL resistance through downregulation of surface expression of death receptors 4 and 5 [36]. Wang's laboratory reported that reduced miRNA MIR23B increases ATG12 and autophagy to promote radioresistance in pancreatic cancer cells [37]. An analysis of the transcriptional status of ATG12 in > 50 breast cancer cell lines suggested that the ATG12 transcript is commonly upregulated in trastuzumab-unresponsive HER2-overexpressing breast cancer cells [38].

Here, we report that 3-BrPA had different effects in two breast cancer cell lines (Figure 1) although autophagy occurred after drug treatment in both lines (Figure 2). Autophagy inhibitors CQ and 3-MA used during 3-BrPA treatment revealed that CQ and 3-MA enhanced 3-BrPA-induced cell death in both cell lines (Figure 3A, 3B and 3C). Then, siRNA-mediated knockdown of Atg7 sensitized cells to 3-BrPA treatment (Figure 3E, 3F). Thus, autophagy played a protective role during 3-BrPA treatment.

Autophagy can prevent apoptosis [39], necrosis [40], and necroptosis [41]. We used z-VAD-fmk to rule out the contribution of pan-caspases in 3-BrPA plus CQ-induced cell death. z-VAD rescued 3-BrPA plus CQ-induced cell death in MDA-MB-435 cells but exacerbated cell death in MDA-MB-231 cells. Furthermore, MDA-MB-231 underwent necrotic cell death after 3-BrPA plus CQ treatment, as evidenced by EM. Thus, necrosis is the predominant mode of cell death in this line. Finally, Nec-1 rescued 3-BrPA plus CQ-induced cell death in MDA-MB-231 and MDA-MB-435 cells. These findings strongly suggest that 3-BrPA plus CQ induce necroptosis in MDA-MB-231 cells. RIPK1 is a multi-functional protein also known to mediate NF-κB and caspase-8 activation in response to TNF-α[42]. The pro-apoptotic function of RIPK1 is the formation of a caspase-8 activation complex in response to TNF receptor I activation [43]. RIPK1 was also reported to be important for triggering necroptosis [44]. In our study, knockdown of RIPK1 rescued both two cells from 3-BrPA plus CQ-induced cell death. In addition, knockdown of other known contributors to programmed necrosis, such as RIPK3, only rescued MDA-MB-231 cells from 3-BrPA-induced cell death. Interestingly, 3-BrPA plus CQ induces apoptosis in MDA-MB-435 cells, but induces necroptosis in MDA-MB-231 cells, and all require RIPK1. These data suggest a potential for novel strategies for the treatment of breast cancer.

Autophagy induction after mitochondrial damage can lead to ROS production [45]. ROS, at the physiological level, are important for a variety of cellular programs of physiological and pathological conditions. However, aberrantly high ROS are intimately associated with disease and are commonly observed in cancer [46]. ROS regulate autophagy by multiple mechanisms that involve direct effects on Atg protein Atg4 [47], affecting the activity of the master autophagy regulator mTORC1 [48]. ROS are also implicated in autophagy induction in cancer therapy [49], suggesting that ROS are important with respect to the mechanism of actions of cancer chemotherapeutics. Deregulation of ROS formation is associated with cancer initiation, progression, and drug resistance. In our study, inhibition of autophagy led to an increase in ROS formation and blocking ROS accumulation with NAC rescued 3-BrPA plus CQ-induced cell death (Figure 5A, 5B, 5C), suggesting that ROS accumulation is an important mechanism in the sensitization of cells to death when autophagy is suppressed.

In summary, 3-BrPA-induced autophagy in breast cancer cells may function as a resistance mechanism against cell death. Then, inhibition of autophagy could be a novel strategy for breast cancer adjuvant therapy. Although detailed mechanisms driving ROS generation and autophagy in our model are unclear, our data will inform future studies in cancer cells treated with 3-BrPA.

## MATERIALS AND METHODS

### Ethics statement

Tumor assays in mice were performed using Institutional Animal Care and Use and proved by the Committee on the Ethics of Animal Experiments of the Bengbu Medical College (Permit Number: 013). Mice were monitored for overall health in response to drug treatment. All efforts were made to minimize suffering during the experiment.

### Materials

Cell culture materials were obtained from Invitrogen and fetal calf serum was from Gibco. 3-bromopyruvate, chloroquine diphosphate, 3-methyladenine (3-MA), 3-(4, 5-dimethylthiazol-2-yl)-2, 5-diphenyltetrazolium bromide (MTT), Nec1 were purchased from Sigma-Aldrich. z-VAD-fmk was purchased from Calbiochem. JC-1 and Annexin V-FICT/PI assay were purchased from KeyGEN BioTECH (China). GFP-LC3 plasmid was purchased from GeneCopoeia. The following antibodies were used: LC3, Bclin-1 (MBL), Bax, Bak, Bcl-2, Mcl-1 (ProteinTech), Atg7 (Beyotime), RIPK1 (Santa cruz), RIPK3 (Cell Signaling).

### Cell Culture

MDA-MB-435 and MDA-MB-231 cells were purchased from Cell Bank (Chinese Academy of Sciences). Cells were grown in DMEM containing 10% FCS, 100 U/ml penicillin, 100 μg/ml streptomycin, and were maintained at 37 °C in a humidified atmosphere of 5% CO_2_ in air.

### SiRNA Design and Validation

siRNA against ATG7 (GGUCAAAGGACGAAGAUAATT UUAUCUUCGUCCUUUGACCTT), RIPK1 (GCAAAGACCUUACGAGAAUTT AUUCUCGUAAGGUCUUUGCTT) and RIPK3 (CCAGUGACGUCUACAGCUUTT AAGCUGUAGACGUCACUGGTT) were designed by Gene Pharma. Knockdown efficiency of individual siRNA was validated by western blot analysis. All siRNAs were obtained from Gene Pharma.

### RNA interference

Cells were grown on 30-mm glass coverslips to 75% confluence and transfected with either siRNA or plasmid using the Lipofectamine™ 2000 transfection reagent from Invitrogen. 70 pmol of the respective siRNA(s) were mixed with transfection reagent in 0.5 ml of opti-MEM (Invitrogen) without FCS and incubated at room temperature for 20 min. Cells were incubated for 24 h and the medium was exchanged with complete culture medium. 48 h post-transfection, cells were incubated with drugs and viability was assessed by MTT assay.

### Plasmid transfection and confocal microscopy analysis

For overexpression of GFP-LC3 cells were transfected with 1 ml of serum-free opti-MEM containing 2 μg of plasmid DNA and 5 μl of Lipofectamine™ 2000. The medium was complemented after 1 h with 1ml of full culture medium. Cells were incubated for 6 h and the medium was replaced by complete culture medium. 48 h post-transfection, cells were incubated with drugs. The treated cells were fixed with 4% paraformaldehyde and examined by a confocal microscopy (Nikon D-Eclipse-C1).

### Electron microscopic detection of autophagosome

Cells were collected and fixed with 3% glutaraldehyde and 2% paraformaldehyde in 0.1 M PBS buffer (pH 7.4) for 30 min, postfixed with 1% osmium tetroxide for 1.5 h, washed and stained in 3% aqueous uranyl acetate for 1 h then dehydrated in an ascending series of ethanol and acetone, and embedded in Araldite. Ultrathin sections were cut on a Reichert ultramicrotome, double stained with 0.3% lead citrate and examined on OLYMPUS JEOL electron microscope.

### MTT Assay

Cells were plated in a 96-well plate and allowed to adhere overnight before drug treatment. After the indicated treatments, cells were incubated with MTT at a final concentration of 0.5 mg/L. After 3-4 h, the medium was removed, and the cells were dissolved in DMSO 150 μL each well and the blue dye was allowed to dissolve for 30 min at room temperature. The absorbance was subsequently measured at 570 nm using a microplate reader (BioTek). Data were normalized to respective controls and represented as percent viability of the controls.

### AnnexinV-FITC/PI Staining

The detection was performed by the AnnexinV-FITC Apoptosis Detection Kit (KeyGEN BioTECH). Briefly, cells were seeded in 6-well plates and incubated for 12 h and then exposed to drugs. After treatment, approximately 1×10^5^ cells were harvested and washed with warm PBS prior to stain with Annexin V-FITC and PI according to the manufacturer's instructions. After 20 min of incubation, cells were analyzed by flow cytometry (BD, Accuri C6).

### Western Blotting

Cells were washed twice with ice-cold PBS and lysed. The protein concentration was measured using a BCA protein assay kit (Beyotime). An equal amount of protein were separated by SDS-PAGE and transferred to PVDF membranes. The membranes were blocked with 5% nonfat dry milk in PBS containing 0.05% Tween 20 (Sigma) for 4 h and then incubated with the primary antibody at 4 °C overnight. The following day, the membrane was incubated with horseradish peroxidase-conjugated secondary antibody for 2 h. The membrane was further developed with the ECL plus western blotting detection system (BioTek).To control the equal amount of protein loading, membrane was reprobed with β-actin antibody.

### Mitochondrial Membrane Potential (MMP) Analysis

The detection was performed by fluorescence microscope using JC-1 staining according to the manufacturer's instructions. Briefly, following drug treatment, cells were incubated with 10 μM JC-1 for 30 min at 37 °C in the dark. Then cells were detected by fluorescence microscope (Olympus, 1X71).

### Measurement of reactive oxygen species (ROS)

Intracellular ROS levels were measured using dihydroethidium (DHE), a cell-permeable indicator for ROS generation. Following drug incubation, 5 μM DHE added to the media and incubated 30 minutes in the dark. Then cells were harvested, washed with PBS and detected by flow cytometry. The results were analyzed by Cell Quest analysis software.

### *In vivo* tumor experiment

The nude mice (5-6 weeks) used in these studies were obtained from Beijing vitalriver and weighed 20–25 g at the time of tumor implantation. The mice were kept under a 12:12 h light–dark cycle, at 24 ± 2 °C and fed with clean food and water. Human MDA-MB-231 cells (107cells/ml) were inoculated subcutaneously to form tumors. Mice with tumors (100-200 mm^3^) assorted to four groups (5 mice/group). Vehicle (0.9% NS) or CQ (40mg/kg/d, 24 days) or 3-BrPA (5mg/kg/d, 24 days) alone or in combination was administrated intraperitoneally. Tumor growth was monitored every three days by two-dimensional measurements of individual tumors for each mouse. Tumor volume was calculated using the formula: length×width^2^/2. After treatment ended, mice from each group were sacrificed. Tumors were excised, calculated and fixed in 4% formalin solution, embedded in paraffin, and then stained with hematoxillin-eosin (H&E).

### Statistical analysis

Statistical analyses were performed with Concise Statistics (CS 14.0) software. The data presented were mean ±standard deviation (SD). Data were compared using Student's t test. *p*< 0.05 was considered significant.
